# Size Dependent Plasmonic Effect on BiVO_4_ Photoanodes for Solar Water Splitting

**DOI:** 10.1038/srep16660

**Published:** 2015-11-19

**Authors:** Liwu Zhang, Lars O. Herrmann, Jeremy J. Baumberg

**Affiliations:** 1 Shanghai Key Laboratory of Atmospheric Particle Pollution and Prevention, Department of Environmental Science and Engineering, Fudan University, Shanghai, 200433 (China); 2 Cavendish Laboratory, Department of Physics, University of Cambridge, Cambridge, CB3 0HE (UK)

## Abstract

Plasmonic nanostructures show great promise in enhancing the solar water splitting efficiency due to their ability to confine light to extremely small volumes inside semiconductors. While size plays a critical role in the plasmonic performance of Au nanoparticles (AuNPs), its influence on plasmon-assisted water splitting is still not fully understood. This holds especially true for low band gap semiconductors, for which interband excitations occur in wavelength regions that overlap with plasmonic resonances. Here, BiVO_4_ films are modified with AuNPs of diameters varying from 10 to 80 nm to study the size dependence of the plasmonic effect. Plasmon resonance energy transfer (PRET) is found to be the dominant effect in enhancing the water splitting efficiency of BiVO_4_. “Hot electron” injection effect is weak in the case of BiVO_4_/AuNP. This is attributed to the interband excitation of BiVO_4_, which is unfavourable for the hot electrons accumulation in BiVO_4_ conduction band. The resonant scattering effect also contributes to the enhanced water splitting efficiency for the larger diameter AuNPs. It is also for the first time found that higher PRET effect can be achieved at larger off-normal irradiation angle.

Artificial photosynthesis, using solar energy to produce hydrogen or other fuels, is a very attractive route to reduce the impact of energy production on climate change and address the increasing global energy demand^1^,^2^,^3^,^4^,^5^. Photoelectrochemical (PEC) water splitting is one of the promising routes for the conversion of solar energy into hydrogen^6^,^7^,^8^,^9^,^10^. The practical application of this technology is hindered by a lack of inexpensive and efficient materials as photoelectrodes. Metal oxides are frequently studied as photoelectrode materials, because they are usually inexpensive, robust and easily fabricated^11^,^12^,^13^,^14^,^15^,^16^,^17^,^18^. However, their solar-to-hydrogen energy conversion efficiency is relatively low. It is still a great challenge to develop efficient PEC materials for water splitting, which involves satisfying multiple requirements.

In a typical PEC water splitting cell, the semiconductor in the photoanode or photocathode converts incident photons to electron-hole pairs. The charge carriers are then separated due to the electric field inside the semiconductor. The photogenerated electrons reduce water to form hydrogen at the cathode, while at the anode the photogenerated holes oxidize water to form oxygen gas. In a PEC cell with high Faraday efficiency of H_2_ and O_2_ production, the water splitting performance can be evaluated through the photocurrent measurements. The low solar-to-H_2_ energy conversion efficiency in metal oxides is attributed to the high bulk recombination rate. The light absorption in a semiconductor can be divided into three spatial regions^19^: i) A space charge layer, in which the photogenerated charge carriers can be separated with the assistance of electric field, ii) a diffusion layer, in which the photogenerated charge carriers can diffuse into the space charge layer and be transported with field assistance, and iii) a bulk layer, where the charge carriers generated recombine before they can reach the surface. The charge carriers generated in the first and second regions can contribute to the photocurrent. Unfortunately, most of the charge carriers are generated in the third region, where they recombine and become useless. It is thus anticipated that higher solar-to-H_2_ energy conversion efficiency can be achieved if one can confine and manipulate light absorption in the first and second regions of the semiconductor. Recently, plasmonic metal nanoparticles have attracted extensive attention in improving solar water splitting efficiency^20^,^21^,^22^,^23^,^24^,^25^,^26^,^27^,^28^,^29^, due to their ability to strongly confine light in the vicinity of their surface.

Plasmonic effects for solar water splitting have been widely studied in recent years^20^,^21^,^22^,^23^,^24^,^25^,^26^,^27^,^28^,^29^. For example, Warren and coworkers observed a relative enhancement in the water splitting activity of α-Fe_2_O_3_ at photon frequencies corresponding to the plasmonic resonance in gold^23^. Thomann *et al.* showed that resonances in plasmonic nanostructures can be engineered to concentrate incident light to the location where the water splitting takes place^24^. A 2.5-fold improvement of AM1.5 photocurrent has been achieved by van de Krol and coworkers via the application of Ag@SiO_2_ core–shell nanoparticles on the surface of BiVO_4_ to improve the absorption^30^. The plasmonic resonance of metallic nanoparticles can be tuned through adjustment of their shapes or sizes in order to match the semiconductor absorption bands^31^. It has recently been reported by Wei and coworkers that the size of gold nanoparticles (AuNP) is essential for efficient “hot electron” injection and plays a critical role in determining the reduction potential of the transferred electrons in the conduction band of TiO_2_^22^. However, the precise influence of AuNP size on plasmon-assisted water splitting is still poorly understood, especially for visible-light-active semiconductors, which have interband excitation in the spectral region enhanced by the plasmonic resonance.

Here, BiVO_4_ films combined with AuNPs with diameters varying from 10 to 80 nm are investigated as photoanodes for PEC water splitting. We observe that the plasmonic field confinement and water splitting efficiency of BiVO_4_/AuNPs sensitively depends on the particle size of the AuNPs. Maximum enhancement is found for AuNPs with a diameter of 30 nm due to a pronounced overlap of the resonantly amplified electric near-field with the interband excitation in BiVO_4_. Finite-difference time-domain simulations reveal a strong dependence of the plasmon resonance energy transfer (PRET) on the diameter of the AuNP. The resonant photon scattering effect only plays an important role when the AuNP size is bigger than 60 nm. The PRET effect is also found to strongly depend on the angle of incidence of light. A higher PRET effect can be achieved at larger off-normal irradiation angle, due to the increased electric field component along the surface normal.

To compare the plasmonic effect of AuNPs with different diameters on BiVO_4_ photoanodes for solar water splitting, commercially available AuNPs with nominal diameters of 10, 20, 30, 40, 60 nm are dropcasted on a film of BiVO_4_. The densities of AuNPs over the BiVO_4_ photoanodes are calculated based on the concentration of these AuNP dispersions in order to maintain a uniform coating density of 1.4 × 10^11^ particles/cm^2^. Figure 1 displays scanning electron microscope (SEM) images for the AuNP modified BiVO_4_ films. The bare BiVO_4_ film is observed to be composed of BiVO_4_ nanoparticles of size from tens of nanometers to 300 nm. For the fabrication of BiVO_4_ film, the precursor dip-coating and annealing process were repeated for three times to get a dense BiVO_4_ film on FTO. Due to this repeated coating process, most of the pores from the removal of the organic components have been eliminated. From the SEM image under higher magnification (see Supplementary Fig. S1 online), it is found that the film is very dense although with a rough surface. Therefore, most of the AuNPs locate on the top surface of BiVO_4_ film, which is also confirmed by the SEM observations. Most of the AuNPs are homogeneously distributed across the BiVO_4_ film, while small amounts of AuNPs aggregate into clusters, especially for AuNPs bigger than 30 nm. The AuNPs in the same image show similar diameters, confirming their uniformity in size.

The photo-electrochemical properties of the bare BiVO_4_ and AuNP modified BiVO_4_ electrodes were studied both in the dark and under simulated solar illumination (100 mW/cm^2^). A conventional three electrode configuration was used with BiVO_4_ as working electrode, Pt wire as counter electrode, and Ag/AgCl as reference electrode. The electrolyte was aqueous phosphate buffer at pH 7 without any additive. The linear voltammetry sweeps (LVS) (scan rate: 10 mV/s) on the bare BiVO_4_ and BiVO_4_/AuNP electrodes under chopped illumination from the back-side of the electrodes are displayed in Fig. 2. All samples show a steady increase of the photocurrent with the applied positive potential, with negligible currents in the dark. Fast and uniform photocurrent responses are observed for each switch-on and switch-off event for all electrodes. The maximum current of the chopped light LVS is approximately linear and is reached instantaneously upon illumination.

All the BiVO_4_ photoanodes modified with AuNPs achieve higher photocurrent densities than bare BiVO_4_ photoanodes, as shown in Fig. 2. Highest photocurrent densities are obtained with 20 nm and 30 nm AuNPs. The same trend is observed in Fig. 3a, which displays LVS (scan rate: 10 mV/s) on the bare BiVO_4_ and BiVO_4_/AuNPs electrodes under constant illumination, as well as in the dark. The dark scan reveals a small background current of 1 μA/cm^2^. The first order derivative of the LVS is plotted in Fig. 3b to show the onset potentials of the photoelectrodes. For the bare BiVO_4_, the anodic photocurrent is observed at more than the open-circuit potential of 0.05 V vs. Ag/AgCl, while in the case of AuNP modified BiVO_4_ photoanodes the onset potentials are slightly moved to more negative potentials (from 0.01 to −0.05 V vs. Ag/AgCl). The onset potential of bare BiVO_4_ is similar to that reported for BiVO_4_ thin films deposited via solution-based methods^32^,^33^,^34^,^35^. Photocurrent densities at 0.6 V vs. Ag/AgCl of various photoelectrodes under illumination from the back-side or front-side of the electrodes were extracted and plotted in Fig. 3c. Since all the BiVO_4_ photoanodes are obtained from the same large piece of BiVO_4_ film, the origin of the error bars (spread of results on different samples) is likely due to the variation of AuNP aggregation on the BiVO_4_, since it is known that the optical properties of AuNPs are modified when dispersed or aggregated in different manners^36^. For all these electrodes, back-side illumination produces higher photocurrent than front-side illumination, which has been reported to be related to the poor electron transport in BiVO_4_^37^. In BiVO_4_, the BiVO_4_ crystal is composed of non-interconnecting VO_4_ tetrahedra (see Supplementary Fig. S2 online). The conduction band of BiVO_4_ consists mainly of V 3d orbitals, consequently, the photogenerated electrons have to hop between VO_4_ tetrahedra in BiVO_4_. In the PEC cell, the photogenerated electrons need to travel from the place generated to the FTO conductive substrate. Under backside illumination most electrons are generated close to the FTO substrate, the required diffuse length is thus shorter than for frontside illumination. In both cases, enhanced photocurrent densities are obtained for all the BiVO_4_ films after addition of AuNPs with diameters between 10 nm and 80 nm. The maximum enhancement factor is reached for 20 nm and 30 nm AuNP modified samples, resulting in a ~100% increase in the photocurrent density both under illumination from back-side and front-side. In the case of back-side illumination, the lowest photocurrent density is recorded for BiVO_4_/AuNP-40 nm, but is still 33% enhanced over that of bare BiVO_4_. As the size of AuNPs further increases to 60 nm, the measured enhancement factor is higher than BiVO_4_/AuNP-40 nm, while the photocurrent density drops again for BiVO_4_/AuNP-80 nm photoanode. For the front-side illumination, the lowest photocurrent enhancement is observed at AuNP-80 nm.

Localized surface plasmon resonance (LSPR) brings several benefits to solar water splitting. First, plasmon resonance energy transfer (PRET) is one of the effects that provide plasmon-enhanced water splitting. The effect of PRET can result in field-enhanced electron-hole production: the interaction of localized electric fields around the plasmonic metal particles with the neighbouring semiconductor allows for the enhanced formation of electron-hole pairs in the near-surface region of the semiconductor. Second, plasmon-induced charge transfer, in which hot electrons excited by the surface plasmons can be injected into the conduction band of the semiconductor, can create additional charge carriers. Third, due to resonant photon scattering at AuNPs, the average photon path-length can be increased.

If the enhanced photoactivity is induced by the effect of PRET, the enhancement should be different due to the shift of SPR absorption wavelength when the diameter of the AuNPs varies. The PRET effect with different diameters of AuNPs can be understood from plots of the electric field distribution in the AuNP modified BiVO_4_ electrodes, which can assess the extent of electric field amplification at the interface region of BiVO_4_ surface and AuNP. We thus performed electric field distribution simulations based on the finite-difference time-domain (FDTD) method. The simulated spatial distributions of the electric field are shown in Fig. 4. Figure 4a depicts the electric field distribution in 10 nm, 20 nm, 40 nm and 60 nm-diameter AuNP modified BiVO_4_ for incident light of 500 nm and 550 nm. The black lines in these graphs show the contour where |E/E_0_| = 1, i.e., the electric field intensity is not amplified. It can be seen that with increasing AuNP diameter, the volume of enhanced electric field also increases. For example, under incident light of 550 nm, the maximum depth in BiVO_4_ with enhanced electric field is 3 nm, 5 nm, 12 nm and 20 nm for AuNPs with diameters of 10 nm, 20 nm, 40 nm and 60 nm, respectively. Under incident light of 550 nm, the electric field intensity at the interface and the surface region of BiVO_4_ near the AuNP is enhanced by 20, 35, 40 and 50 times for AuNPs with diameters of 10 nm, 20 nm, 40 nm and 60 nm, respectively. The electric field intensity at the surface region of BiVO_4_ is also enhanced by more than 5 times even under incident light of 500 nm. While BiVO_4_ possesses a hole diffusion length of 100–200 nm^34^, the photogenerated holes in these regions with enhanced electric field can readily diffuse to the surface of BiVO_4_. Photogenerated charge carrier separation can benefit from this localized light absorption in the surface region of BiVO_4_, where the charge carriers can be separated with the assistance of electric forces in the space charge layer.

Apart from the increased volume of enhanced electric field in BiVO_4_, the enhancement factor is also higher when the diameter of AuNPs is larger. Figure 4c illustrates the field enhancement at a point which is 2 nm below the interface of AuNP and BiVO_4_ as a function of incident light wavelength. It is found that the enhancement of the electric field reaches a maximum at around 580 nm for all the AuNPs. The field enhancement becomes more pronounced as the size of AuNPs increases. A two fold field enhancement at a wavelength of 580 nm is observed for 10 nm-diameter AuNPs, while a nearly 20-fold enhancement is achieved for AuNPs with diameter of 60 nm. From Fig. 3c, we find that the photocurrent increases as the diameter of AuNPs increases from 10 nm to 30 nm. Based on these simulations, we attribute this to the effects of increased volume of enhanced electric field and higher enhancement factors in BiVO_4_. The localized light absorption and enhanced light absorption on the surface of BiVO_4_ due to the plasmonic antenna effect of AuNPs can further facilitate charge carrier generation in the space charge layer of BiVO_4_ which are readily separated.

The measured enhancement factor is however less pronounced when the diameter of AuNPs further increases beyond 30 nm (Fig. 3c), which seems to contradict the electric field simulation results. One reason for this is a reduction in the spectral overlap between the BiVO_4_ light absorption band and the red-shifting plasmonic absorption of AuNPs with increasing size, which hampers the effectivity of PRET. Figure 5a shows the plasmonic absorption of AuNPs with different diameter and also the absorption edge of the BiVO_4_ photoanode. A red shift of the plasmonic absorption peak from 528 nm to 549 nm is observed on increasing the AuNP diameter from 10 nm to 60 nm. The extinction spectra of the BiVO_4_/AuNP films are shown in Supplementary Fig. S3. A schematic drawing of the difference between the absorption and scattering efficiency of the AuNP as well as the extinction measurement (Fig. S3) can be found in Supplementary Fig. S4. However only plasmonic absorption shorter than the BiVO_4_ absorption edge (530 nm) can enhance the photoactivity through PRET. Therefore, a further increase in the size of AuNPs reduces the effect of PRET. Another reason for the reduced enhancement with larger diameter AuNPs is the reduction of exposed area of BiVO_4_ to electrolyte, which can influence the water oxidation efficiency on the BiVO_4_ surface. Since all the BiVO_4_/AuNP samples possess the same density of AuNPs, a much larger area on the BiVO_4_ is covered by larger diameter AuNPs, as can be seen in the SEM images of BiVO_4_/AuNP (Fig. 1). With a AuNP density of 1.4 × 10^11^ particles/cm^2^, 15% of BiVO_4_ surface area is covered with 60 nm AuNPs, while the value is only 0.4% for 10 nm AuNPs. It is well known that surface area plays a critical role in the photocatalytic water splitting process. Therefore, less exposed surface area of BiVO_4_ due to the increased coverage with larger AuNPs can be another reason for the weaker photocurrent enhancement.

Figure 5b shows the action spectra of bare BiVO_4_ and BiVO_4_/AuNP photoanodes. Compared with bare BiVO_4_, the BiVO_4_/AuNP photoanodes show an increased response at wavelengths longer than the absorption edge of BiVO_4_ (530 nm), although very weak. This response is attributed to the plasmon-mediated electron transfer from AuNP to BiVO_4_. Electrons in the AuNP can be promoted to high energy by the photoexcited plasmons, facilitating a transfer of electrons from the surface plasmon states to the conduction band of the semiconductor. This “hot electron” injection mechanism rather than PRET effect is frequently proposed to be the main reason for plasmon-assisted TiO_2_ water splitting^38^,^39^,^40^,^41^,^42^,^43^, as TiO_2_ has no interband excitation in the wavelength region that is enhanced by the plasmonic resonance. However, here we find that the “hot electron” injection effect is weak for the case of BiVO_4_/AuNP. This is attributed to fact that the interband excitation of BiVO_4_, with a light absorption edge of 530 nm, directly overlaps with the plasmonic resonance. Under irradiation, electrons are already excited to the conduction band (CB) of BiVO_4_, which can reduce the Schottky barrier at the interface of AuNP and BiVO_4_. It is known that the Schottky barrier helps the transferred “hot electrons” to accumulate in the CB of semiconductor, preventing them from traveling back to the AuNP^22^. Therefore here, the interband excitation of BiVO_4_ is responsible for the weak “hot electron” injection in BiVO_4_/AuNP. It is thus proposed that the PRET effect is the main reason for the enhanced photocurrent in the case of BiVO_4_/AuNP photoanodes. This is also confirmed by studying the enhancement factor of action spectra after the combination of BiVO_4_ with 20nm or 30 nm AuNPs (Fig. 5c,d). We observe a good match between the enhancement in the action spectra and the plasmonic absorption of the AuNPs. The broad light absorption of BiVO_4_ enables field-enhanced electron-hole production due to the plasmonic resonance. To eliminate the influence of incident light intensity, the incident photon-to-electron conversion efficiency (IPCE) spectra of bare BiVO_4_ and BiVO_4_/AuNP photoanodes are further shown in Fig. 6.

In the case of back-side illumination, when the diameter of AuNPs reaches 60 nm, the photocurrent enhancement is higher than that of 40 nm AuNPs (Fig. 3b). This increase is ascribed to the resonant photon scattering effect of AuNPs, which can increase the path length of light in the BiVO_4_ film and enhance the light absorption in the film, as shown in Fig. 7a. We thus studied the resonant scattering effect of AuNPs with different size through Mie Theory calculations (Fig. 7b). It is observed that the scattering of AuNPs smaller than 40 nm is very weak, whereas it is much stronger in the case of 60 nm AuNPs. Therefore, for the BiVO_4_ photoanode modified with 60 nm AuNPs the resonant scattering effect should be taken into account, and is thought to be responsible for the increase in photocurrent compared to 40 nm AuNP modified BiVO_4_. However, when the diameter of the AuNPs increases to 80 nm, no further photocurrent enhancement due to the resonant photon effect is observed (Fig. 3b), but a reduction in the photocurrent is found as a result of weakened PRET and unfavourable coverage effects as discussed above. It is worth noting that the intensity of scattered light on the AuNP is identical in the forward and reverse directions. The intensity, *I*, of the scattered radiation is given by


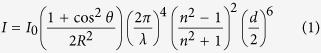


where *I*_0_ is the light intensity before the interaction with the particle, *R* is the distance between the particle and the observer, *θ* is the scattering angle, *n* is the refractive index of the particle, and *d* is the diameter of the particle. The intensity of scattered light is proportional to (1+cos^2^ *θ*), indicating that the intensity of scattered radiation is identical in the forward and reverse directions. However, in the case of front-side illumination, the intensity of light scattered into the environment is stronger than that of back-side illumination (*I*_0_ is higher in front-side illumination), resulting in a more efficient light utilization in the case of back-side illumination, which could be another reason for the higher photocurrent response in this case.

Angle-dependent measurements were performed to further confirm the PRET effect between AuNPs and BiVO_4_ photoanode. Electric field distribution simulations by FDTD show that the electric field amplification near the AuNP sensitively depends on the irradiation angle of the light source, as illustrated in Fig. 8a–c. These show the electric field enhancements under irradiation with off-normal angle varying from 45 to 90^o^ on 20 nm, 40 nm and 60 nm sized AuNP modified BiVO_4_, respectively. It is found that for these AuNP diameters, the electric field enhancement at the location 2 nm below the interface of AuNP and BiVO_4_ tends to become larger as the off-normal angle increases. This is due to the increased electric field component along the direction of the surface normal which leads to stronger near-field enhancement underneath the particle. Correspondingly, the photocurrent is expected to vary with the light irradiation angle, if the PRET effect exists in the AuNP modified BiVO_4_ photoanode system. Figure 8d shows the angle-dependent photocurrent enhancement on BiVO_4_ with and without AuNPs at 0.6 V vs. Ag/AgCl. The reported photocurrents in this experiment are corrected by taking into account reflection loss of light at larger off-normal irradiation angle. No significant change is observed on the photocurrent measured on BiVO_4_ with an increasing off-normal irradiation angle. However, for the BiVO_4_ photoanode modified with 20 nm AuNPs, the photocurrent response is increased by more than 30% as the off-normal angle shifts from 0^o^ to 75^o^. The photocurrent at an off-normal angle of 75^o^ is 2.5 times higher than bare BiVO_4_. This enhancement in photocurrent at high off-normal irradiation angle on AuNP modified BiVO_4_ is attributed to the stronger plasmonic electric field enhancement at high off-normal angle, as shown in the electric field simulations in Fig. 8a. For instance, at a wavelength of 525 nm, the simulations predict an electric field enhancement at off-normal angle of 75^o^ that is almost twice as high as that under 45^o^ angle of incidence. This increased enhancement at larger off-normal angle can be explained by the simulation of the field profile as a function of angle of incidence, which is plotted in Fig. 8e. It shows that as the axial component of the electric field vector increases, the substrate coupled mode is excited more efficiently and therefore more of the field is localized underneath the particle, within the BiVO_4_. This explains the increase in field enhancement as the angle of incidence increases.

It is notable that the AuNPs (especially larger particles) are faceted and do not resemble perfect spheres. As such, they may have much larger contact area with the substrate. Therefore, we further did simulations for a 60 nm spherical AuNP and a faceted one (icosahedral in shape) under 60° angle of incidence plane wave illumination. Shown in Fig. 9 is the resonant near field enhancement. The local maximum enhancement is lower in the faceted case. However the overall volume is slightly enhanced and the absorption resonance is broadened. Furthermore, strong enhancement occurs at the edges of the facets which remain accessible for water. In the contact region, it may be difficult for water to penetrate. These PRET modifications and larger contact area in the case of faceted Au nanoparticles could also contribute to the reduced photocurrent enhancement at larger AuNPs.

To verify that the measured photocurrent of the BiVO_4_/AuNPs photoanodes originates from water splitting rather than any other undesired side reactions, a water splitting experiment was performed at 0.5 V (*vs*. Ag/AgCl), and the gas evolution were measured (Fig. 10). The ratio of evolution rates of H_2_ and O_2_ with the BiVO_4_/AuNP-30 nm as photoanode is close to the stoichiometric value of 2.0, with rates of 13.5 μmol/h/cm^2^ for H_2_ and 6.6 μmol/h/cm^2^ for O_2_. The faradaic efficiencies for both gases are higher than 95%, suggesting that the observed photocurrent can be fully attributed to water splitting. The photocurrent slightly decreases by less than 10% after 4 hours water splitting, implying the high stability of the BiVO_4_/AuNPs photoanodes. The gas evolution over BiVO_4_/AuNP-10 nm and BiVO_4_/AuNP-60nm are also investigated and shown in Fig. 10. The gas evolutions over these samples resemble that of BiVO_4_/AuNP-30 nm, but the evolution rate is lower, which is consistent with the photocurrent measurements.

In summary, the size dependent effect of plasmon-assisted water splitting on BiVO_4_ has been investigated both experimentally and theoretically. We observe that the size of AuNPs plays a critical role in plasmonic BiVO_4_/AuNP photoanodes for solar water splitting. Due to the interband excitation of BiVO_4_ in the wavelength region which is enhanced by the plasmonic resonance, PRET is the dominating effect in enhancing the water splitting efficiency of BiVO_4_. AuNPs with diameters larger than 40 nm reduce the PRET effect due to the red shift of the plasmon absorption resonance. The PRET effect is found to be angle-dependent, and stronger PRET is observed at larger off-normal irradiation angles. The overall photocurrent enhancement is expected to be even higher if the absorption band of BiVO_4_ matches the field enhancement. Future work will be done to extend the light absorption of BiVO_4_ by doping to better match the field enhancement. The work here provides a better understanding of the effect of AuNP size in plasmon-assisted water splitting, especially for visible-light-active semiconductors which have interband excitations in the light region directly enhanced by the plasmonic resonance of the nanoparticles. A better understanding of the mechanism of plasmon-assisted water splitting can thus help developing more efficient solar-to-fuel energy conversion systems.

## Methods

### Synthesis of BiVO_4_/AuNP photoanodes

The amorphous complex precursor used for the fabrication of the BiVO_4_ films is produced as follows: 0.02 mol of diethylenetriaminepenta-acetic acid (H_5_DTPA) and 7.5 mL ammonia in water (13.0 mol/L) were added to 200 mL hot distilled water. After dissolution, 10 mmol of Bi(NO_3_)_3_ (Sigma-Aldrich) and 4.850 mmol of V_2_O_5_ powder (Sigma-Aldrich) were added. The resulting mixture was stirred and heated at 80 °C to promote complexation of Bi^3+^ and V^5+^ with DTPA and formation of a transparent solution.

Dip-coating method is employed to coat the BiVO_4_ precursor onto the surface of FTO conductive glass. Then BiVO_4_ is formed from the precursor and crystallized by heating at 500 °C for 3 hours in air. This dip-coating and annealing process were repeated for three times to get a dense BiVO_4_ film on FTO. Commercially available AuNPs with sizes of 10, 20, 30, 40, 60, 80 nm (British Biocell International) were drop cast onto the top surface of BiVO_4_ electrode, and the sample kept in a 40 °C oven until dry. The required volume of AuNP dispersion was calculated based on the concentration of these AuNP dispersions to keep the coating density at a constant value of approximately 1.4E11 particles/cm^−2^. In order to precisely investigate the effect of AuNPs with different diameters, and remove the influence of variation in experimental conditions for BiVO_4_ film fabrication, in this work a large piece of BiVO_4_ film on FTO is first prepared, which is then cut into small pieces for investigating size dependent effects of AuNPs on BiVO_4_ films. This method avoids variations in the deposition process and ensures that the BiVO_4_ photoanodes studied are identical.

### Photoelectrochemical (PEC) measurement

Photoelectrochemical measurements were carried out on an electrochemical workstation (IVIUMSTAT). A conventional three electrode configuration was used with BiVO_4_/AuNP, Pt wire and Ag/AgCl electrode as working electrode, counter electrode and reference electrode, respectively. The electrolyte was an aqueous phosphate buffer at pH 7. The working electrodes were illuminated from the back side unless otherwise noted. The light source is a 100 mW/cm^2^ Xenon light source (Bentham IL75E). For action spectra measurements a Bentham DTMc300 monochromator was used. The evolved gas was quantitatively analyzed using a gas chromatograph (Shimadzu 8A, TCD detector). The GC was equipped with a molecular sieve 5 A packed column.

### Material Characterizations

Scanning Electron Microscope (SEM) measurements were performed on a LEO GEMINI 1530VP FEG-SEM.

### Simulations

Electromagnetic simulations were carried out using the finite-difference time-domain method (Lumerical FDTD v8.6). The AuNP was illuminated with a p-polarised plane wave. The dielectric function of Au was taken from Johnson and Christy^44^ and a refractive index of 2.5 was assumed for BiVO_4_^45^.

The reflection loss associated with irradiation angle is given by these equations:

For light at an angle of incidence *θ*_0_ and angle or refraction *θ*_1_ in the two media, the reflectance *R* depends on the state of polarization of the light and is given by


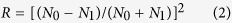


where for light polarized with the electric vector parallel to the plane of incidence (p vibration, TM wave).


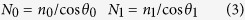


while for light with the electric vector perpendicular to the plane of incidence (s vibration, TE wave)


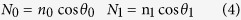


where *n*_0_ is the refractive index of water and *n*_1_ is the refractive index of BiVO_4_. In this work, the incident light is unpolarized, so the reflectance is the mean of *R*_p_ (*R* of p vibration) and *R*_s_ (*R* of s vibration).

## Additional Information

**How to cite this article**: Zhang, L. *et al.* Size Dependent Plasmonic Effect on BiVO_4_ Photoanodes for Solar Water Splitting. *Sci. Rep.* **5**, 16660; doi: 10.1038/srep16660 (2015).

## Supplementary Material

Supplementary Information

## Figures and Tables

**Figure 1:**
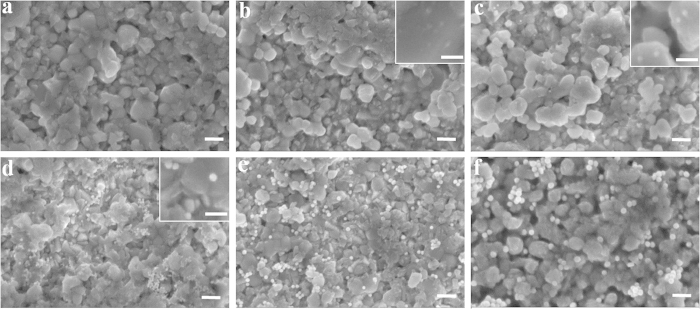
SEM images: (**a**) Bare BiVO_4_ film. (**b**) BiVO_4_/AuNP-10 nm. (**c**) BiVO_4_/AuNP-20 nm. (**d**) BiVO_4_/AuNP-30 nm. (**e**) BiVO_4_/AuNP-40 nm. (**f**) BiVO_4_/AuNP-60 nm. Scale bar is 200 nm. Scale bars in the insets are 100 nm.

**Figure 2:**
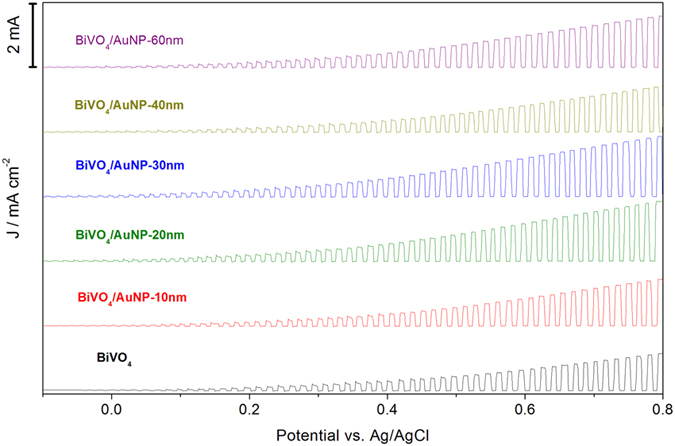
Linear voltammetry sweeps under chopped illumination (back-side) on bare BiVO_4_ and BiVO_4_ modified with AuNP photoelectrodes of increasing diameter.

**Figure 3:**
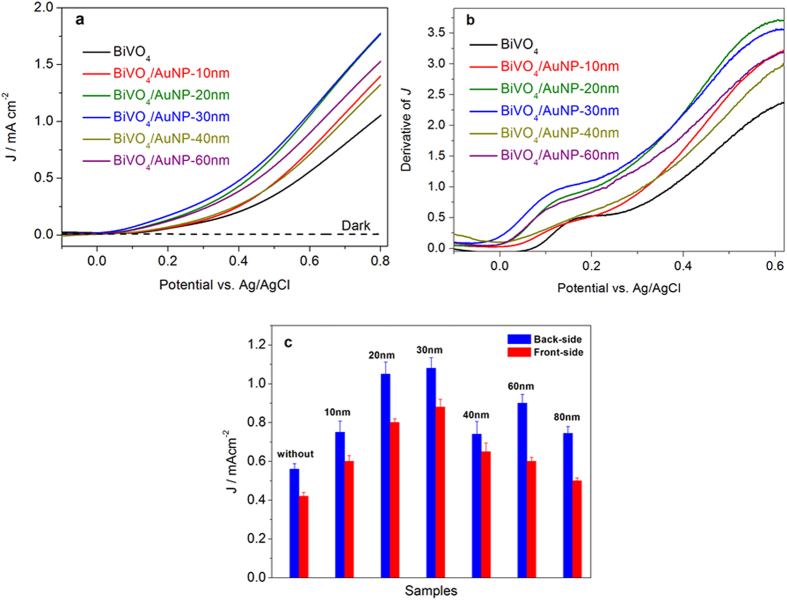
(**a**) Linear voltammetry sweeps on bare BiVO_4_ and BiVO_4_/AuNP photoelectrodes. (**b**) First order derivative of the linear voltammetry sweeps. (**c**) Photocurrent densities at 0.6 V vs. Ag/AgCl of BiVO_4_ and BiVO_4_/AuNP photoelectrodes, under illumination from back-side or front-side of the electrodes.

**Figure 4:**
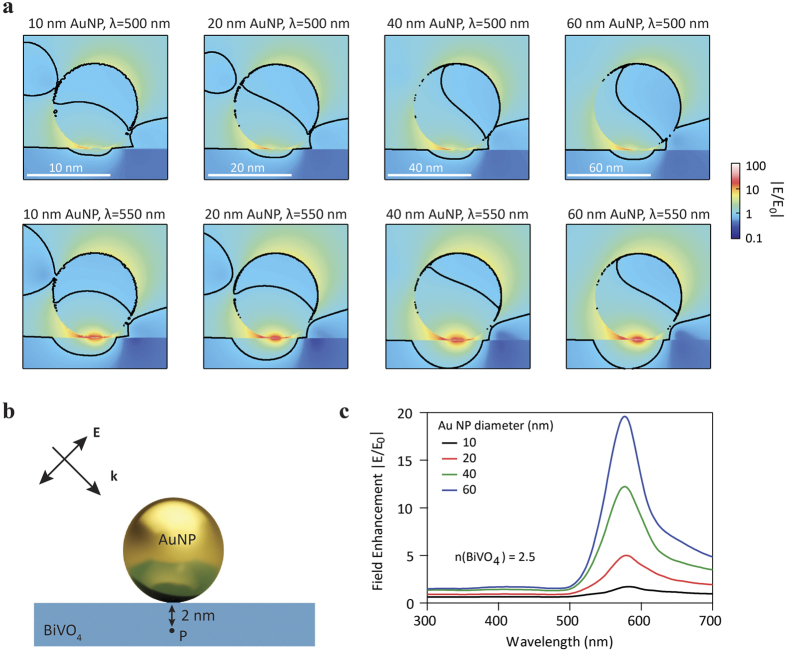
Finite-difference time-domain (FDTD) simulations. (**a**) Electric field distribution in 10 nm, 20 nm, 40 nm and 60 nm-diameter AuNP modified BiVO_4_ for incident light of 500 nm (top) and 550 nm (bottom). (**b**) Model used for the simulations. (**c**) Field enhancement at position 2 nm underneath the interface of AuNP and BiVO_4_, as a function of incident light wavelength (extracted from the simulation).

**Figure 5:**
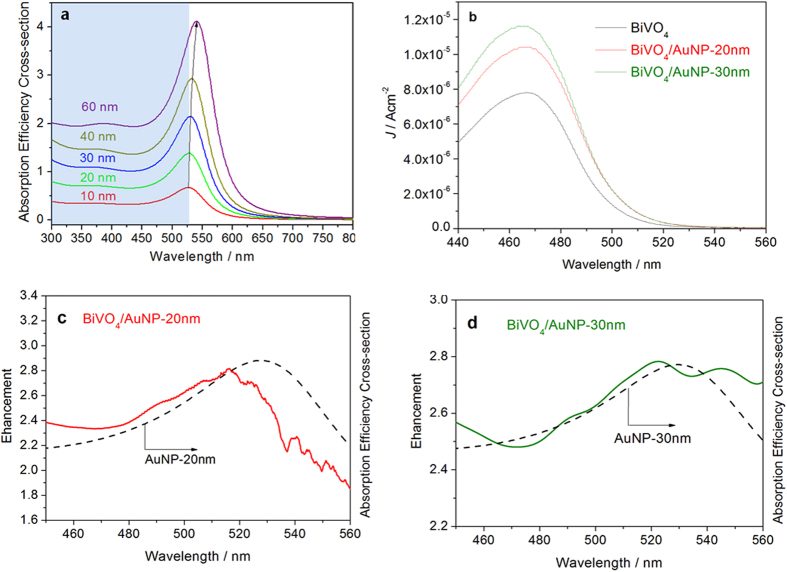
(**a**) Plasmonic absorption of AuNPs with different diameter. The absorption edge of BiVO_4_ photoanode occcurs at around 530 nm (blue shaded). (**b**) Action spectra of bare BiVO_4_ and BiVO_4_/AuNP photoanodes. (**c**) Action spectra enhancement factor of BiVO_4_/AuNP-20 nm photoanode, with dashed line showing plasmonic absorption of 20 nm AuNPs. (**d**) Action spectra enhancement factor of BiVO_4_/AuNP-30 nm photoanode, with dashed line showing plasmonic absorption of 30 nm AuNPs.

**Figure 6:**
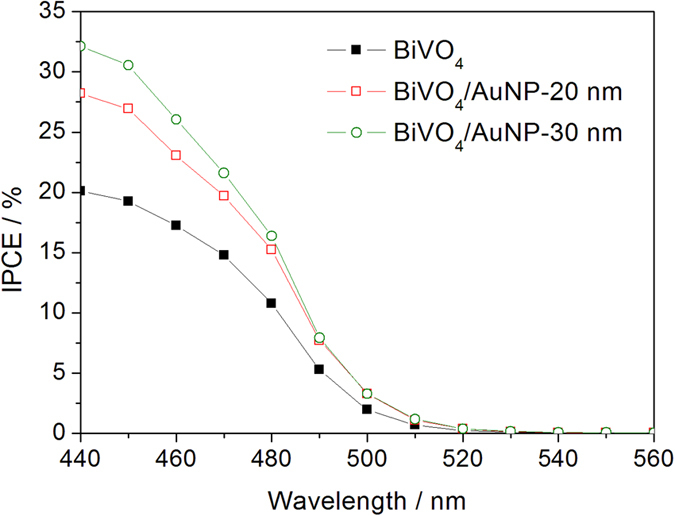
IPCE spectra of bare BiVO_4_ and BiVO_4_/AuNP photoanodes.

**Figure 7:**
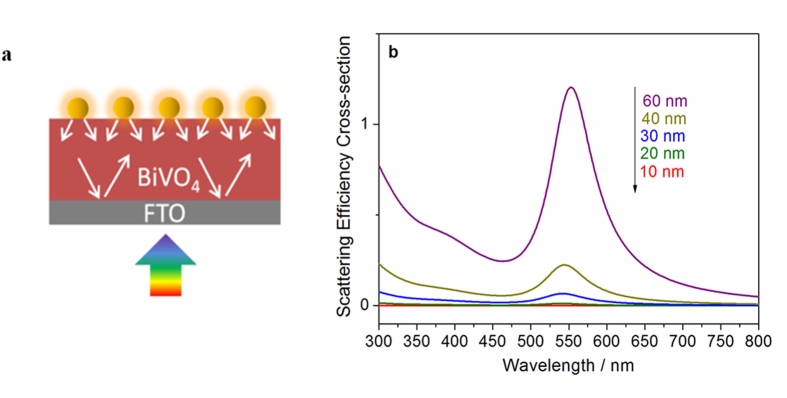
(**a**) Illustration of resonant photon scattering effect of AuNPs, which can increase the path length of light in the BiVO_4_ film. (**b**) Resonant scattering effect of AuNPs with different size obtained from Mie Theory calculations.

**Figure 8:**
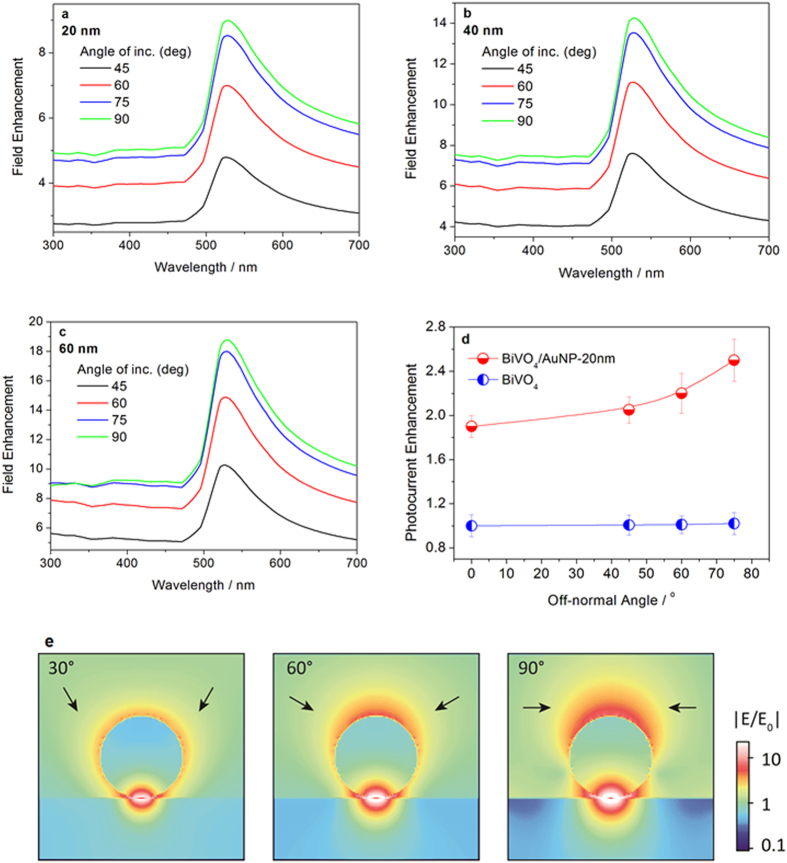
Angle-dependent electric field enhancement at a point 2 nm below the interface of the AuNP and BiVO_4_ for diameters of (**a**) 20 nm, (**b**) 40 nm and (**c**) 60 nm, respectively. (**d**) Angle-dependent photocurrent response on bare BiVO_4_ and BiVO_4_/AuNP-20 nm at 0.6 V vs. Ag/AgCl. (**e**) Electric field distribution in 60 nm-diameter AuNP modified BiVO_4_ under irradiation with off-normal angles of 30^o^, 60^o^ and 90^o^.

**Figure 9:**
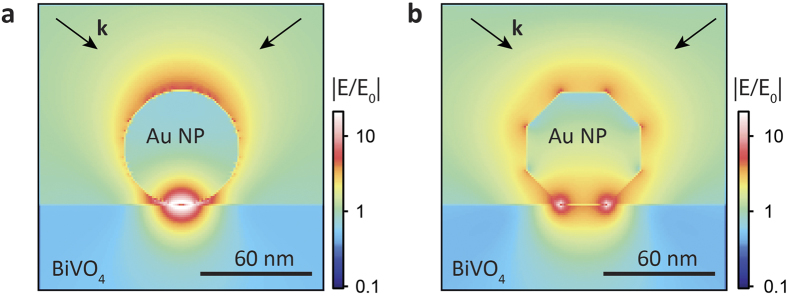
Electric field distribution on a 60 nm spherical AuNP (**a**) and a faceted one (icosahedral in shape) (**b**) under 60° angle of incidence plane wave illumination.

**Figure 10:**
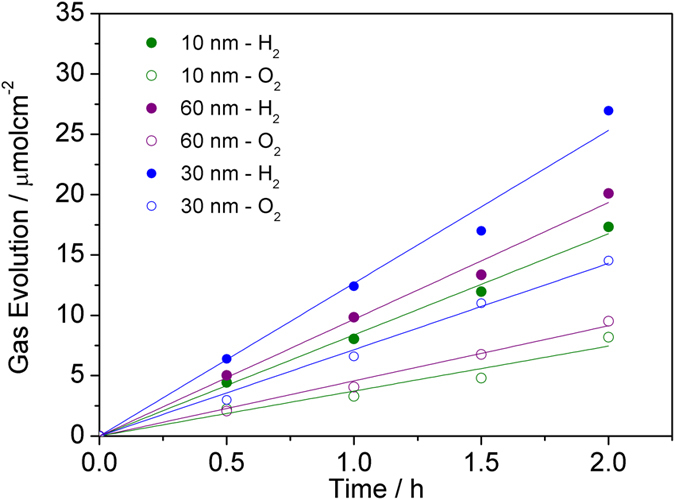
Gas evolution at an applied potential of 0.5 V (*vs*. Ag/AgCl).
