# Supplementary material for: Size Dependent Plasmonic Effect on BiVO4 Photoanodes for Solar Water Splitting
Source: Sci Rep. 2015 Nov 19;5:16660. doi: 10.1038/srep16660 (PMC4652227; doi:10.1038/srep16660)
Supplement: Supplementary Information [file srep16660-s1.pdf]

Supporting Information

Size Dependent Plasmonic Effect on $BiVO_4$ Photoanodes for Solar Water Splitting

Liwu Zhang,*[a,b] Lars O. Herrmann[b] and Jeremy J. Baumberg*[b]

[a]Shanghai Key Laboratory of Atmospheric Particle Pollution and Prevention, Department of Environmental Science and Engineering, Fudan University
Shanghai, 200433 (China)
E-mail: zhanglw@fudan.edu.cn
[b]Cavendish Laboratory, Department of Physics, University of Cambridge
Cambridge, CB3 0HE (UK)
E-mail: jjb12@cam.ac.uk

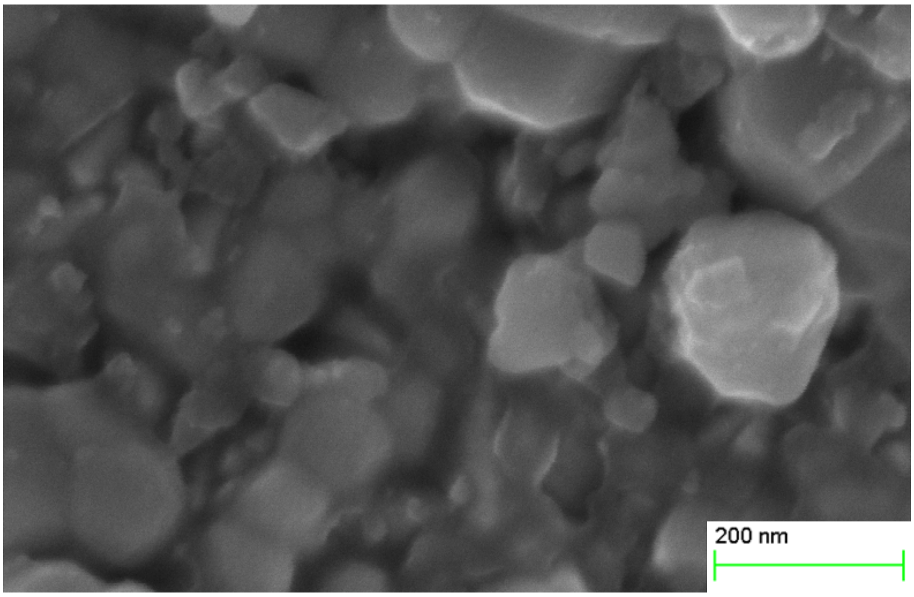

Figure S1. SEM image of $BiVO_4$ film under higher magnification.

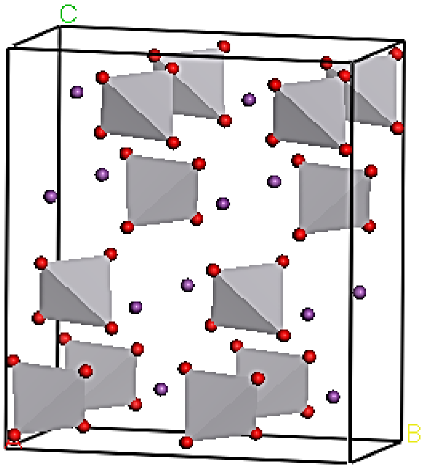

Figure S2. Crystal structure of $BiVO_4$, purple ball represents Bi and red ball is O.

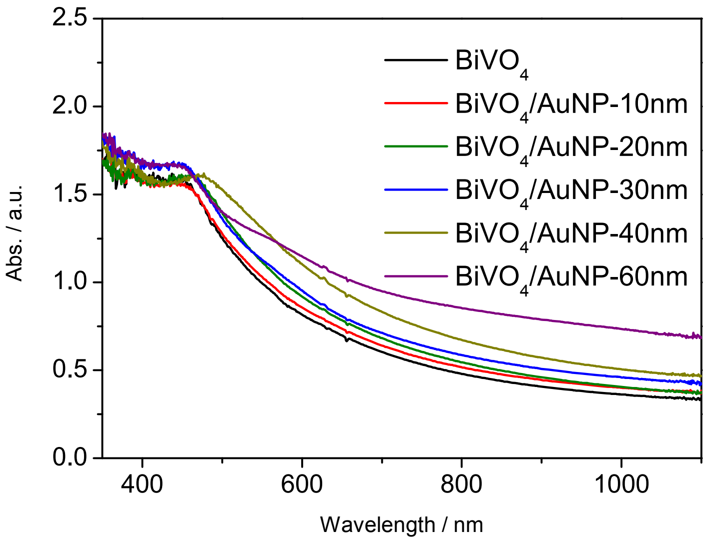

Figure S3. Extinction spectra of $BiVO_4$/AuNPs photoanodes.

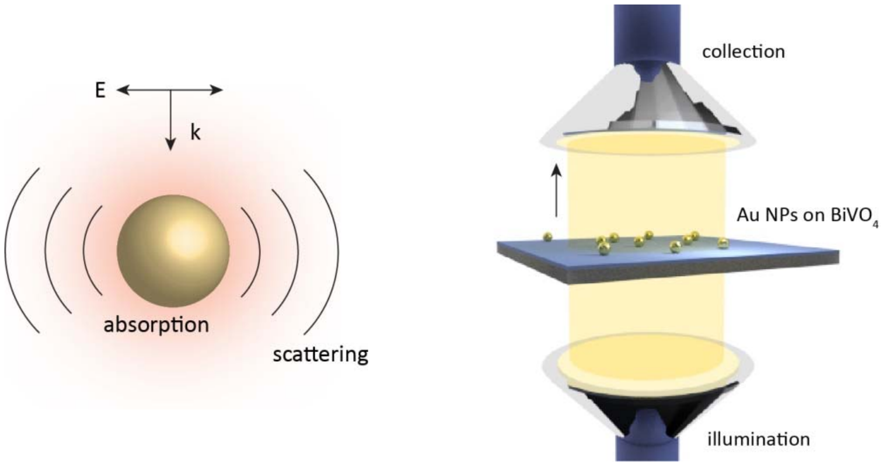

Figure S4. A schematic drawing of the difference between the absorption and scattering efficiency of the AuNP (in Figure 5a and 7b) as well as the extinction measurement of $BiVO_4$/AuNP(in Figure S3).

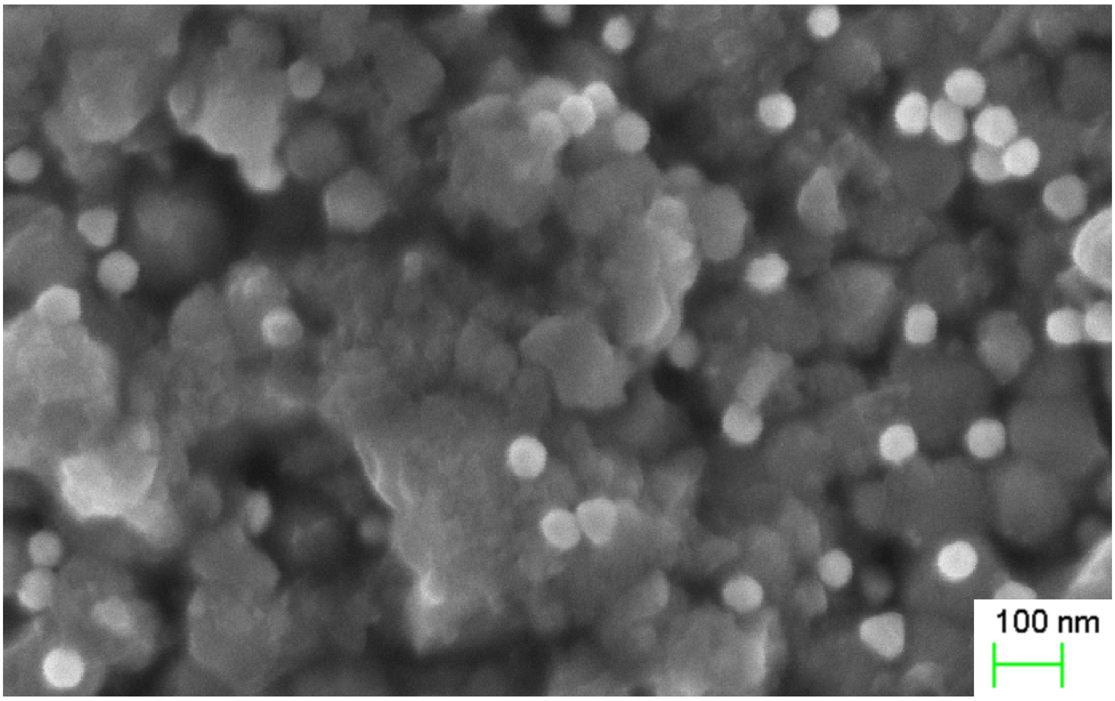

Figure S5. SEM image of $BiVO_4$/AuNP film under higher magnification.
